# Cell Death Is Counteracted by Mitophagy in HIV-Productively Infected Astrocytes but Is Promoted by Inflammasome Activation Among Non-productively Infected Cells

**DOI:** 10.3389/fimmu.2018.02633

**Published:** 2018-11-20

**Authors:** Diego S. Ojeda, Daniel Grasso, Javier Urquiza, Andreas Till, María Inés Vaccaro, Jorge Quarleri

**Affiliations:** ^1^Consejo Nacional de Investigaciones Científicas y Técnicas (CONICET), Instituto de Investigaciones Biomédicas en Retrovirus y Sida (INBIRS), Facultad de Medicina, Universidad de Buenos Aires, Buenos Aires, Argentina; ^2^Consejo Nacional de Investigaciones Científicas y Técnicas (CONICET), Instituto de Bioquímica y Medicina Molecular Departamento de Fisiopatología, Facultad de Farmacia y Bioquímica, Universidad de Buenos Aires, Buenos Aires, Argentina; ^3^Institute of Reconstructive Neurobiology, University of Bonn, Bonn, Germany; ^4^Life and Brain GmbH, Bonn, Germany

**Keywords:** HIV, reservoirs, central nervous system, astrocytes, mitochondria, inflammasome, autophagy, mitophagy

## Abstract

Despite more than 30 years of extensive research efforts, a complete understanding of the neurological consequences of HIV central nervous system (CNS) infection remains elusive. HIV is not only able to establish a viral reservoir in the CNS but also to initiate manifestation of neurodegenerative diseases. These neurological disorders may arise because of virus-induced activation of the inflammasome in CNS cells, including astrocytes. Nevertheless, in some productive viral infection scenarios, selective autophagy may reduce inflammation through mitochondrial degradation (“mitophagy”) to counteract inflammasome activation. In this study, using cultured human astrocytes, we demonstrate that–depending on the HIV infection outcome–cells may resist death, or succumb by inflammasome activation when viral infection is productive or abortive, respectively. Cells productively infected with HIV were able to attenuate both mitochondrial ROS production and mitochondrial membrane potential dissipation, thus exhibiting cell death resistance. Interestingly, mitochondrial injury was counteracted by increasing the autophagic flux and by activating mitophagy. Conversely, astrocytes exposed to HIV in an abortive scenario showed prominent mitochondrial damage, inflammasome activation, and cell death. This bystander effect occurred after cell-to-cell contact with HIV-productively infected astrocytes. In summary, we demonstrate a tight functional crosstalk between viral infection mode, inflammasome activation, autophagy pathways and cell fate in the context of HIV infection. Moreover, mitophagy is crucial for cell death resistance in HIV-productively infected astrocytes, but its impairment may favor inflammasome-mediated cell death in abortively infected cells.

## Introduction

In the central nervous system (CNS), where resident lymphoid cells are absent, human immunodeficiency virus (HIV) infection is restricted to microglia and astrocytes (1). Astrocytes are part of the blood-brain barrier and are the most abundant cell type in the brain. *In vivo*, HIV can infect these cells, establish latency and replicate without cytopathic effects facilitating the viral invasion into the CNS (2–4). Raised levels of cellular oxidative stress, immune activation and inflammation are mechanisms contributing to HIV-associated neurocognitive disorders (HAND) (5). In this scenario, astrocytes sense damaged- and pathogen-associated molecular patterns (DAMPs and PAMPs, respectively) via Nod like receptors (NLRs) which, among other pathways, control inflammasome activation through post-transcriptional mechanisms (6). NLRP3 inflammasome activation is a two-step process involving at first priming of the pathway by enhancing levels of NLRP3 and pro-IL-1β, and then triggering the activation of caspase-1 and consequent processing and release of mature IL-1β (7, 8). Inflammasome activation is a principal contributor to promote glial activation, cytokines production, and to cooperate in neuronal damage during viral CNS infections (9, 10). Previous reports have demonstrated HIV-mediated triggering of the NLRP3 inflammasome in the CNS and subsequent release of the proinflammatory cytokines IL-1β and IL-18 (11–14). Therefore, whereas the capacity of HIV infection to induce NLRP3 inflammasome activation has been well described, the precise molecular mechanism(s) underlying HIV-mediated NLRP3 activation remains poorly understood.

Mitochondria perform diverse yet interconnected functions critical for cellular survival while also contributing to cellular stress responses, e.g., by induction of the intrinsic mode of programmed cell death. Mitochondrial-derived reactive oxygen species (mROS) have been considered to represent one of the major endogenous DAMPs capable to activate the NLRP3 inflammasome under cellular stress conditions (15).

Mitochondrial dysfunction has emerged as a key factor in several infectious diseases -including those with viral etiology (16)- as a critical hub for the regulation of the innate antiviral immune response (17). Surplus or damaged mitochondria can undergo tightly controlled turnover through a selective type of autophagy termed “mitophagy” (18). It is considered a homeostatic mechanism that preserves a healthy mitochondrial population thus playing a cytoprotective role in the context of viral infections and limiting the detrimental effect of mitochondrial damage to avoid the inflammasome activation (19, 20).

The present study addresses new and divergent relationships between cells productively infected with HIV and neighboring non-productively infected and/or uninfected astrocytes. Former cells display prominent mitophagy as a critical strategy to subvert imminent cell death due to increased mitochondrial injury. In contrast, those astrocytes that suffer from this bystander effect experienced NLRP3 inflammasome activation related to mitochondrial damage. These opposite scenarios on modulation of mitochondrial dynamics have an impact on cell viability and consequently influence viral persistence in the brain.

## Materials and methods

### HIV infection of cultured human astrocytes

Human astrocytes were challenged with pseudotyped HIV co-expressing G glycoprotein from stomatitis vesicular virus (VSV-G) (21, 22). We used pNL4.3, a full-length infectious molecular clone of HIV, obtained through the NIH AIDS Reagent Program (Division of AIDS, NIAID, NIH, USA) from Dr. Malcolm Martin (23). The NL4.3-eGFP molecular clone contains the enhanced version of green fluorescent protein gene (eGFP) and an internal ribosome entry-site (IRES) inserted upstream from the functional nef reading frames in the NL4-3 clone. Nef expression is driven by an IRES element between the reporter gene and nef. The pNL4.3-eGFP vector was used to engineer the pNL4.3-DsRed. In brief the eGFP from pNL4.3-eGFP was replaced with a corresponding fragment of the *Discosoma sp*. red fluorescent protein from pCAG-DsRed (Clontech) using *NcoI* and *XmaI* restriction sites. VSV-G pseudotyping of envelope defective viruses was performed by cotransfection of 293T cells with a VSV-G expression plasmid (pCMV–VSV-G) at a HIV/VSV-G plasmid ratio of 10:1. Then, 24 h later, medium was replaced, and supernatants containing lentiviral particles were collected at 48 and 72 h after transfection, pre-cleared by centrifugation, ultra-concentrated over 5 h at 18,000 rpm; the pellet was resuspended in DMEM supplemented with 10% fetal bovine serum (FBS) and stored at −86°C until use.

Normal human astrocytes (NHA) (Lonza®, Pharma&Biotech-Bioscience Solutions) were employed. NHA were seeded in 50 ml tissue culture flasks (5,000 cells/cm2) and, following manufacturer's instructions, were grown in AGM™ Bullet Kit™ medium (Lonza®) at 37°C and with 5% CO_2_). Culture medium was replenished every 2 days, and cells were subcultivated after reaching 85% confluence. After removal of the medium, and washing with serum-free medium, the cells were used in the assays described below.

For infection, astrocytes were seeded in a 24 well culture plate at 50,000 cells/well. The following day, cells were infected at defined inoculums with virus stocks containing 100ng/μl of p24 antigen. After 18 h of exposure to virus at 37°C, cells were washed three times with phosphate buffered saline (PBS) to remove the unabsorbed inoculums and incubated in fresh culture medium at 37°C. To determine whether HIV replication correlates with GFP or DsRed expression in astrocytes, we performed a time-course analysis following infection of astrocytes with either HIV-GFP or HIV-DsRed. Three different variables were monitored as a function of time: (1) HIV capsid protein p24 in cell culture supernatants (2) intracellular expression of p24, and (3) HIV gene expression by GFP or DsRed measurement. The peak of expression coincided with the peak of p24 in supernatants following infection with either GFP- or DsRed-expressing virus. Therefore, cell fluorescence as a reflection of GFP- or DsRed-expression allows the identification of productively infected cells in a heterogeneous population comprising both neighbor non-productively infected and/or uninfected (NPI/U), and productively infected cells (PI). Therefore, this experimental system allowed us to evaluate concomitantly a well-defined phenomenon (e.g., apoptosis, ROS production, mROS production, and mitophagy) in both HIV-infected cells and bystander cells.

### Flow cytometry analysis (FACs)

This technique enables to study different populations of cells simultaneously. Following infection with the GFP- or DsRed-expressing viruses, the specific fluorescence of GFP/DsRed was measured upon excitation at 488 nm. For cell death assay, cells were washed with phosphate-buffered saline and subsequently labeled as described below. Labeled cells were analyzed by flow cytometry using a FACSCanto flow cytometer (BD Biosciences). Cells were gated on the basis of side scatter and forward scatter for debris exclusion, subsequently; infected cells were identified by their green or red fluorescence and evaluated by a different cell death assay as explained below. Data from 5 × 10^4^ cells were collected, stored, and analyzed with FlowJo X software (TreeStar). For cell enrichment, productively and abortively (and non-infected) HIV-infected astrocytes were sorted with a FACSAria FUSION (BD Bioscences) based on their GFP fluorescence at 3 days post-infection.

### Western blot

Cells (5 × 10^5^) were lysed in 20 mM HEPES (pH 7.5) buffer containing 0.5% Nonidet P-40, 50 mM KCl, 150 mM NaCl, 1.5 mM MgCl_2_, 1 mM EGTA, and protease inhibitors. The samples were resuspended in SDS-PAGE loading buffer and heated at 95°C for 5 min. Equal amounts of protein were separated by gel electrophoresis using a polyacrylamide gel and transferred to PVDF 0.2 μm membrane by Western Blotting. The membrane was blocked with 5% fat-free dry milk and then incubated with primary antibodies, followed by incubation with secondary antibody and detection by SuperSignal West Pico PLUS (Thermo Fisher Scientific).

### Fluorescence confocal microscopy

Cell images by confocal imaging of fixed cells were captured with a Zeiss LSM 800 laser scanning confocal microscope equipped with a Zeiss AxioObserver Z1, a 63 × /1.4 Plan-Apochromat oil immersion objective and diode lasers as excitation light source. The laser lines used were 405 nm (DAPI), 488 nm (GFP) and 594 nm (Alexa 594). Emitted light was collected through Variable Secondary Dichroics (VSDs) onto a GaAsP-PMT detector. Images were acquired using Zen Blue software (Zeiss) and processed on AxioVision 4.2 software (Carl Zeiss).

### Cell death detection by annexin-V/7-AAD staining

To determine the percentage of early apoptotic cells, phosphatidylserine (PS) cell translocation and plasma membrane permeability were evaluated by dual staining with PE or, APC-conjugated annexin-V and 7-AAD using the Annexin-V/7-AAD apoptosis detection kit (BD Biosciences) and analyzed by flow cytometry using a FACSCanto (BD Biosciences). The percentage of Annexin-V+/7-AAD- cells (representing early apoptotic cells), and annexin-V+/7-AAD+ cells (mostly representing necrotic cells) was determined.

### Measurement of mitochondrial membrane potential gradient (Δψm)

Mitochondrial damage may be measured as dissipation of the mitochondrial membrane potential (Δψm) that is related to programmed cell death activation. Δψm was monitored by selective accumulation of tetramethyl rhodamine methyl ester (TMRM, Thermo Fisher Scientific), a cell-permeable, cationic, red-orange fluorescent dye that accumulates in active mitochondria with intact membrane potentials, or MitoTraker™ Deep Red (MTDR, Thermo Fisher Scientific). TMRM (500 nM) or MTDR (100 nm) were added to cells 15 min before the analysis by FACS.

For real-time evaluation of the mitochondrial membrane potential Δψm and membrane permeability, dual staining with the TMRM or MTDR and Annexin V/7-AAD were evaluated by flow cytometry using a FACSCanto (BD Biosciences). Emission of TMRM was measured in the PE channel and the mitochondrial depolarization was calculated by measuring the decrease in the fluorescence intensity ratio.

### Detection of cellular reactive oxygen species (ROS) generation

Given that oxidative stress has been linked to HIV replication (24) and HIV exposure in glial cells (25, 26), we next aimed to elucidate whether oxidative stress after HIV exposure would impinge differentially on programmed cell death susceptibility according to infection outcome.

#### DCFDA assay

Cellular ROS production (including hydroxyl, peroxyl and other ROS) were measured using a DCFDA assay kit (Abcam) according to the manufacturer's protocol. DCFDA (2',7'-dichlorofluorescein diacetate), a cell-permeable fluorogenic dye, is deacetylated by cellular esterases to a non-fluorescent compound, and later oxidized by ROS to highly fluorescent 2',7'-dichlorofluorescein (DCF), which measures hydroxyl, peroxyl, and other ROS activities within the cell. DCF is a highly fluorescent compound which can be detected by fluorescence spectroscopy with maximum excitation and emission spectra of 495 nm and 529 nm, respectively. After 3 days post-infection, cells were washed twice with PBS, then incubated with 25 μM DCFDA in essential medium at 37°C for 45 min and then evaluated by flow cytometry using a FACSCanto (BD Biosciences).

#### DHE assay

Cellular ROS production, specifically superoxide, was measured using a DHE (dihydroethidium) assay kit (Thermo Fisher Scientific) according to the manufacturer's protocol. It exhibits blue-fluorescence in the cytosol until oxidized, where it intercalates within the cell's DNA, staining its nucleus a bright fluorescent red. After 3 days post-infection, cells were washed twice with PBS, then incubated with 5 μM DHE in essential medium at 37°C for 30 min and then evaluated by flow cytometry using a FACSCanto (BD Biosciences).

### Detection of mitochondrial reactive oxygen species (ROS) generation

Considering that mitochondria are start point for the intrinsic pathway of cell death activation as well as the primary source for ROS production in cells (27), we pursued to elucidate their participation in the HIV-infection outcome. Mitochondrial ROS detection was measured by FACS in cells stained by 5 μm MitoSOX™ (Thermo Fisher Scientific) during 30 min. This fluorescence assay uses a positively charged probe able to accumulate rapidly in mitochondria. Once inside the mitochondria it can be oxidized by reactive species (including superoxide) to ethidium that subsequently binds to DNA to produce fluorescence.

### Assessment of autophagy and mitophagy

Because autophagy is an important response to cellular stress, we next sought to determine whether HIV infection alters autophagy in astrocytes. Likewise, mitophagy is an essential cellular homeostatic mechanism that regulates tightly the number and quality of mitochondrial biomass by targeting ROS-producing mitochondria during cellular stress, limiting inflammasome activation (28, 29).

#### Measurement of autophagy-related markers (LC3, p62)

LC3 (Microtubule-associated proteins 1A/1B light chain 3) is a primarily cytosolic soluble protein. During execution of macro-autophagy (hereafter “autophagy”) this cytosolic form (“LC3-I”) is proteolytically processed and conjugated with phosphatidyl-ethanolamine to generate LC3-II that is recruited to autophagosomal membranes, a conversion process that is used as a monitoring system for autophagy (30). Here, LC3 status was simultaneously measured by three different technical approaches at 3 days post HIV exposure. First, Cytofix/Cytoperm (BD Biosciences) treated human astrocytes were labeled with anti-LC3 (1:300, Cell Signaling #cat D11) plus a secondary fluorescent antibody (Alexa Fluor® 647; Jackson ImmunoResearch, cat#111606047), and used for immunofluorescence analysis. For quantification of the number of endogenous LC3 dots per cell, a total of 50 cells per condition (*n* = 5) were recorded and analyzed using the NIS Element Basic Research software (Nikon®). LC3 punctate with a diameter between 0.2 and 2 μm were counted as positive.

Second, the conversion from the LC3-I soluble form to the LC3-II lipidated form was evaluated using western-blot, and measured as LC3-II/LC-3-I ratio by densitometry.

Third, autophagic activation patterns were measured as fold-change in LC3-II mean fluorescence intensity (gMFI-LC3) by flow cytometry analysis. For this goal, soluble LC3-I was depleted from cells by 30 min treatment with saponin extraction (Perm/Wash, BD Biosciences), so the total fluorescence represents only LC3-II (30). Histogram plots of cell populations were employed in order to quantify autophagic rate.

In addition, the total protein level of autophagy cargo p62/sequestosome 1 (SQSTM1) was measured. The p62 protein levels are often inversely correlated with activity of the autophagy system considering that it is degraded by autophagy as it physically links ubiquitinated proteins to the autophagic machinery to allow their degradation in the lysosome. Since p62 accumulates when autophagy is inhibited, and decreased levels can be observed when autophagy is induced, p62 may be used as a marker to study autophagic flux (31). Even more, a recent study suggested that p62 is recruited to damaged mitochondria that release mROS to regulate negatively NLRP3-inflammasome activation (19). The expression level of SQSTM1/p62 was measured by western blot using a primary antibody (1:2000, Cell Signaling, cat#3868).

#### Measurement of mitophagy

Next, using different techniques, we analyzed whether HIV productive infection stimulates mitophagy. First, the fusion of mitophagosomes with lysosomes whereby degradation and recycling of cargo ensues was measured (32). To this aim, we used a tandem-tagged RFP-EGFP chimera carrying a mitochondrial targeting signal sequence fused in-frame with the RFP and EGFP genes, that reports the exposure of mitochondria to the lysosomal environment by taking advantage of the differential stability of RFP and GFP at low pH (33). Specifically, GFP fluorescence is quenched while RFP remains stable at low pH in the lysosomal lumen, such that intact mitochondria emit fluorescence both in red and green channels (resulting in yellow signal), while mitochondria associated with lysosomes are red fluorescent only. For this goal, astrocytes were transfected with mitophagy reporter plasmid pAT016 (34) using X-tremeGENE^TM^ HP DNA Transfection Reagent (Sigma Aldrich) at day 0 and infected with HIV pNL-4-3 on day 1 (or left uninfected). At 3 days post infection, cells were washed with PBS and fixed in 4% PFA/PBS. Nuclei were stained using DAPI (Thermo Fisher Scientific). Where mentioned, autophagy/mitophagy inhibitors were used: At 3 dpi cells were exposed to the following compounds for 4 h: 3-methyladenine (3-MA, 5 μm; an inhibitor of the phosphatidylinositol 3-kinases or PI3K), mitochondrial division inhibitor-1 (Mdivi-1, a chemical inhibitor of the mitochondrial fission protein Drp1-, 100 μm; Sigma Aldrich), and lysosomal inhibitor chloroquine (CQ, 50 μm; a lysosome/autophagy inhibitor that raises the lysosomal pH thus inhibiting autophagosome-lysosome fusion; Sigma Aldrich). Quantitative image analysis of mitophagy was performed using the CellProfiler software (35) and handmade analysis pipelines (see below).

#### Analysis of mitochondrial dynamics

Mitochondria are dynamic organelles which undergo both the union by fusion and division by fission. Mitochondrial fission facilitates the segregation of dysfunctional mitochondria which are subsequently targeted for mitophagy (36). In contrast, when mitochondrial dynamics are aberrant, the fusion rate is increased, a process accompanied by high ROS accumulation and cell death. In this study, such mitophagic flux was analyzed using two alternative strategies. First, using pAT016 reporter plasmid and image analysis where the following pipeline was applied: 1-identification of nuclei using blue (DAPI) channel; 2-identification of cytoplasm and transfected cells using red fluorescence (cut-off intensity > 0.01); 3-measurement of red and green fluorescence intensity in transfected cells; 4-calculation of ratio red/green, cells displaying red/green ratio > 2.5 were defined as cells undergoing mitophagy, and the percentage of cells undergoing mitophagy was expressed as a function of infection time. For quantitation of mitochondrial mass per cell, MitoTracker™ Red signal intensity in HIV infected (i.e., GFP expressing) or uninfected cells was quantified and adjusted to cell number by normalizing to DAPI intensity. Secondly, morphological changes among mitochondria were visualized by its staining with Alexa Fluor® 488 anti-Cytochrome c (1:50, BD Pharmingen^TM^, cat# 560263) and nuclei stain DAPI (250 ng/ml; Invitrogen) as counterstain, both applied for 20 min in culture medium. For quantitative analysis of mitochondria fragmentation, MitoTracker Green staining was carried out, and membrane potential dissipation was also concomitantly measured using MitoTracker™ Deep Red (MTDR, 100 nM) staining in either HIV infected (DsRed expressing) or uninfected (DsRed negative) cells by FACS. Co-localization of mitochondria with lysosome was analyzed by pixel intensity spatial correlation analysis (Image J) between red mitochondria (stained with MTDR, 100 nM) and green lysosome (stained with LysoTracker™ Green, 200 nM).

Moreover, mitochondrial morphology changes and mitophagy were also examined using transmission electron microscopy (TEM). Briefly, control (i.e., non-exposed) and infected cells grown in 10 cm dishes were washed and fixed with fixative containing 2% glutaraldehyde in 0.1 M sodium cacodylate buffer, pH 7.4. Cell pellets were embedded in 2% agarose, post-fixed with 1% osmium tetroxide, and dehydrated with an acetone series. Samples were infiltrated, embedded in Durcupan and polymerized at 60°C for 48 h. Ultrathin sections were prepared and examined using a JEOL 1,200 EX II transmission electron microscope at 80 kV.

### Lentivirus-delivered small harpin RNA (shRNA) targeting parkin

Parkin is an ubiquitin E3 ligase that plays an important role in the mitochondrial quality control and mitophagy. This protein mediates the engulfment of damaged mitochondria by autophagosomes which facilitate lysosomal degradation (37–39). In order to diminish the Parkin-dependent mitophagy, the expression of Parkin gene (PRKN, formerly known as PARK2) was knocked-down using specific oligonucleotides (5′-GAAUACAUUCCCUACCUCAdTdT-3′, 5′-GGCGCUAUUUGGCGCUUCAdTdT-3') as small interfering RNA (shRNA) that were delivered into the cells by lentiviral like particles (VLPs). For this goal, shRNAs were cloned into the mammalian lentiviral vector pLKO.1 puro (a gift from Bob Weinberg; Addgene plasmid # 8453) and then transfected in HEK293T. Briefly, 2.5 × 10^3^ HEK293T were seeded on a flat-bottom 96-well plate and transfected with a mix of 100 ng pCMV-dR8.91, 100 ng pLKO.1 coding for PARK2 shRNA (named as “Parkin-/-”) or, alternatively, for “scramble” non-targeting shRNA control (named as “Scr”), and 10 ng pCMV–VSV-G per well, using X-treme GENE HP DNA transfection reagent (Roche), following the manufacturer's recommendations. Then, 24 h later, medium was replaced, and supernatants containing lentiviral particles were collected at 48 and 72 h after transfection, pre-cleared by centrifugation, aliquoted, and stored at −80°C.

In order to knock-down the Parkin gene in cultured astrocytes, a total of 3 × 10^4^ cells were transduced with lentiviral vectors by spinoculation (2,200 rpm, 90 min, 37°C) in the presence of 8 μg/ml of polybrene. Forty-eight hours later, transduced cells were selected by the addition of puromycin (3 μg/ml) for 2 days and the used for subsequent experiments.

### Assessment of inflammasome assembly and activation

Previous studies demonstrated that HIV infection promotes inflammasome activation (11–14). Besides, rapid changes in mitochondrial mROS production and membrane potential are critical for inflammasome activation when stimulated with ATP, dsDNA, or specific mitochondria destabilization agents (28, 29, 40).

To examine whether HIV-induced mitochondrial damage is able to prime and promote inflammasome activation, PI- and NPI/U cells were treated with carbonyl cyanide m-chlorophenyl hydrazone (CCCP; 50 μm, 30 min). It is a protonophore used to promote ΔΨm dissipation enhancing inflammasome activation.

To determine inflammasome activity, astrocytes were exposed to ATP (2.5 mM, 30 min) in order to trigger NLRP3 activation. Then, sorted astrocytes (BD FACSAria^TM^ FUSION) were lysed. Protein extracts were immunoblotted with anti-NLRP3 (1:500, Abcam, cat#AB214185), anti-caspase-1 (1:500, Abcam, cat#EPR4321), and anti- Gasdermin D, (GSMD 1:300, Proteintech, Cat#66387).

Additionally, caspase-1 activation was analyzed among cell lysates and culture supernatants from different conditions that included HIV-infected astrocytes, and Parkin silenced HIV-infected astrocytes. Besides, such Parkin knockdown cells were pretreated to generate two different conditions including: (i) 48 h treatment with a mitochondria-targeted antioxidant with scavenging properties for mROS (10 μm, MitoTEMPO, Sigma-Aldrich, cat# SML0737) thus protecting against oxidative damaging to the mitochondria, and (ii) 48 h treatment with a caspase-1 inhibitor (50 μM, Z-YVAD-FMK, Abcam, cat#ab141388).

### Evaluation of cell-to-cell contact as condition for bystander effects

In order to evaluate the ability of HIV infected-astrocytes and its soluble mediators released to induce bystander effects on uninfected cells (with, or without cell-to-cell contact), three different conditions were evaluated. (i) Cell culture supernatant from HIV-infected astrocytes was collected at 2 dpi. Uninfected astrocytes were exposed to this “conditioned media” during 2 days. Conditioned media was used as 1 and 20x concentration. (ii) Co-culture without cell-to-cell contact was assayed using Transwell® system. The inserts are made of a polycarbonate membrane in polystyrene plastic holders with 0.4 μm diameter and pore size 0.6 μm. The upper chamber containing astrocytes exposed to HIV during 2 days are further stand with uninfected astrocytes (bottom chamber) during other 2 days. (iii) Co-culture with cell-to-cell contact was assessed by adding uninfected astrocytes in a 2 dpi HIV-infected cell-culture. In order to distinguish both cellular populations, uninfected astrocytes (targeted for bystander effect) were stained with Violet Cell Proliferation Dye (VCP, CellTrace™, Thermo Fisher Scientific) prior to be added, and then cocultured during 2-days for cell-to-cell contact. Thus, “bystander” astrocytes (VCP stained cells) were differentiated, and HIV-productive infection was measured (GFP) for each cell population using FACS.

Astrocytes from the three conditions were evaluated for both cell membrane permeability (IP) and mitochondrial membrane depolarization (MTDR).

### Statistical analysis

Where applicable, the two-sided Student's *t*-test was used to determine statistical significance. A *P*-value of < 0.05 was considered significant (^*^), *P* < 0.01 highly significant (^**^) and *P* < 0.001 (^***^). For multiple comparisons an ANOVA analysis was performed.

## Results

### HIV infected astrocytes are resistant to mitochondria-triggered cell death

**Figure 1:**
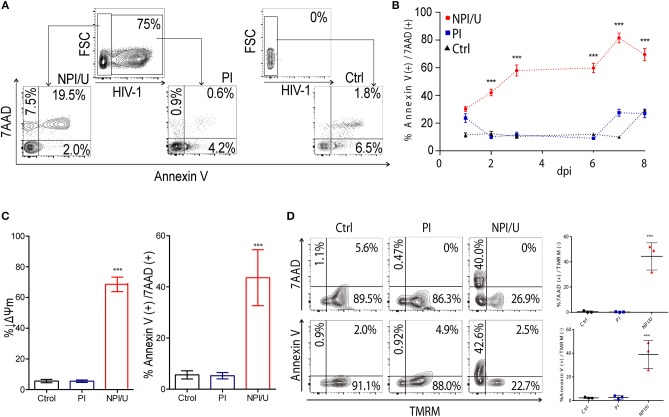
Cell death analysis by FACS according to HIV-infection outcome. **(A)** Representative FACS contour plots showing the relative percentage of cell death between PI and NPI/U astrocyte at 3 days post infection. Data are representative of 3 independent experiments. **(B)** Cell death levels in astrocytes according to HIV-infection progress (expressed as days post-infection/dpi) analyzed by FACS. Black dots: mock infected cells (Control); blue dots: PI cells; red dots: NPI/U cells. ^***^p < 0.0001 NPI/U vs. Ctrl, Student's *t*-test. **(C)** Measurement of early and intermediate stages of cell death by FACS. Diminished mitochondrial membrane potential (↓*Δψ*m) and positive staining for annexin-V and 7-AAD are shown according to cell death percentage. *n* = 3 samples/condition. ^***^p < 0.0001 NPI/U vs. Ctrl, Student's *t*-test. **(D)** FACS contour plots from a representative experiment showing for each scenario (control, PI, NPI/U) a concomitant analysis of the mitochondrial membrane potential dissipation, 7-AAD, and annexin V staining at 3 dpi. The x axis corresponds to fluorescent signal intensity of TMRM, and the y axis shows 7-AAD or Annexin-V fluorescence intensity. Graphics are showing values obtained from 3 independent experiments, small horizontal lines indicates the mean±SD. ^***^p < 0.0001 NPI/U vs. Ctrl, Student's *t-*test.

In order to evaluate cell death during HIV infection, annexin-V and 7-AAD levels were measured by FACS. The efficiency of infection at 3 days post infection (dpi) rose to 65 ± 5% using a GFP-expressing pseudotyped-HIV, allowing to distinguish two cellular scenarios after HIV exposure: (i) human astrocytes productively infected (“PI”) with HIV, and (ii) non-productively infected and/or uninfected (“NPI/U”) neighboring astrocytes (Figure 1A). Programmed cell death levels in NPI/U astrocytes were significantly higher than PI population (Figures 1B,C) at 3 dpi. Non-productively infected astrocytes revealed a plasma membrane pore, as evidenced in co-staining annexin-V and 7-AAD, suggesting necroptotic and/or pyroptotic cell death. This profile persisted on later times during viral infection at 6, 7, and 8 dpi (Figure 1B).

Mitochondrial damage was measured as dissipation of mitochondrial membrane potential (Δψm) at 3 dpi using FACS measurement of tetramethyl-rhodamine methyl ester (TMRM). A marked mitochondrial membrane depolarization (Δψm loss) was observed in NPI/U astrocytes. In contrast, a small proportion among PI and control astrocytes displayed Δψm dissipation (Figure 1C).

To determine whether mitochondrial membrane depolarization and programmed cell death activation were mechanistically related in individual NPI/U astrocytes, both parameters were measured simultaneously by FACS (Figure 1D). More than half of the NPI/U cells exhibiting Δψm dissipation were also 7-AAD positive, further supporting cell death induction. In contrast, similarly to control cells, mitochondria membrane depolarization was minimal among the few PI astrocytes annexin-V/7-AAD positive, suggesting that both mitochondria damage and cell death are scarce and are uncoupled among each other (Figure 1D).

In summary, these data suggest that astrocytes exposed to HIV may be differentially affected by programmed cell death according to the outcome of viral infection. Hence, when HIV exposure results in productive infection, resistance to cell death was observed. In contrast, when the HIV exposure does not cause productive infection, bystander cell death is favored involving mitochondrial damage.

### HIV infection increases cellular reactive oxygen species (ROS) levels in astrocytes

**Figure 2:**
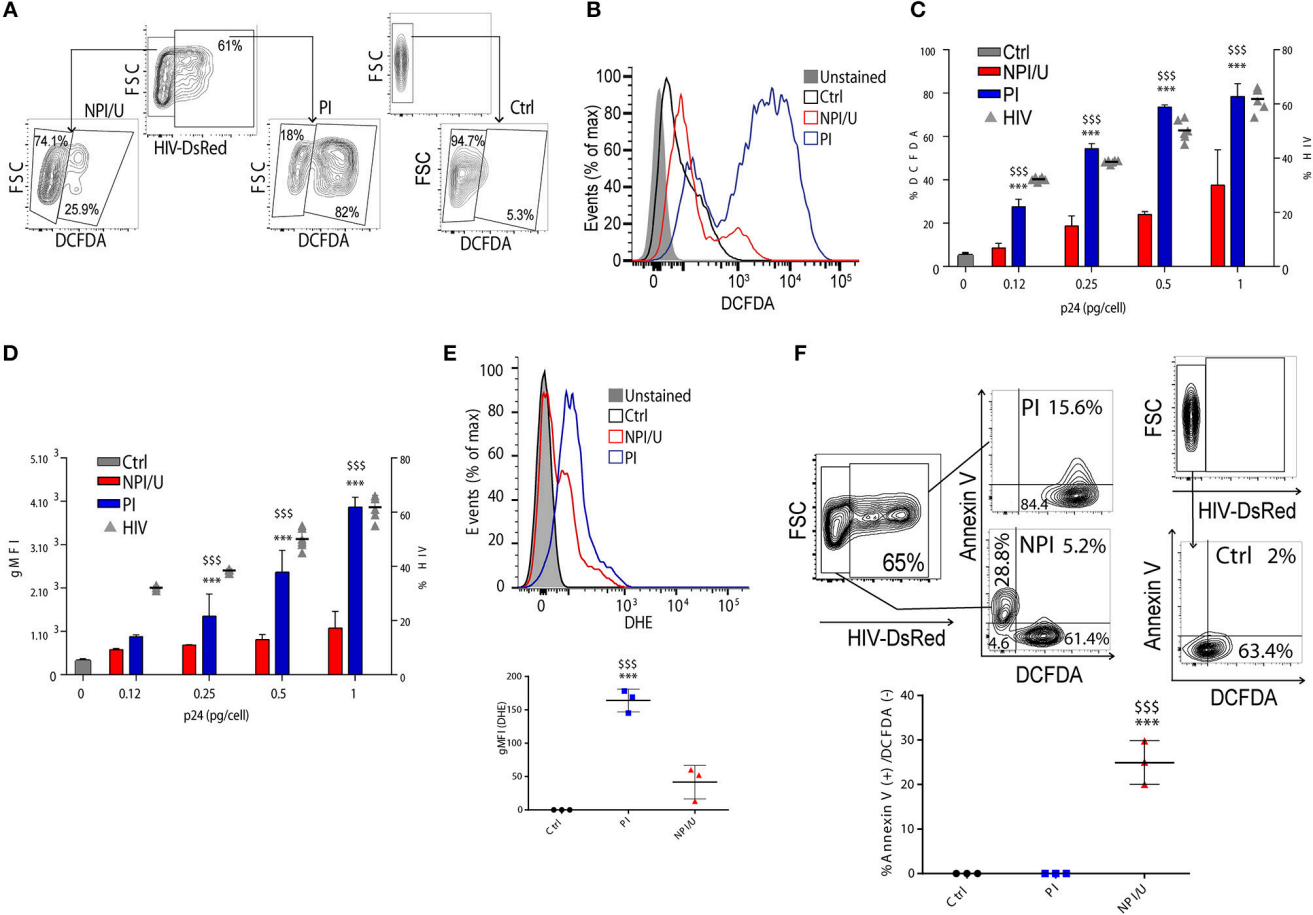
Analysis of ROS production among HIV-productively infected (PI) and non-productively/uninfected (NPI/U) astrocytes. **(A)** FACS contour plots from a representative experiment showing relative percentage control, PI, and NPI/U astrocytes producing ROS, measured using DCFDA at 3 dpi. Graphics are showing values obtained from 3 independent experiments. **(B)** Histogram showing ROS generation at 3 dpi measured by FACS using DCFDA. The x axis represents the fluorescent signal intensity, and y axis the cell number normalized as a percentage of the maximum. Graphics are showing values obtained from 3 independent experiments. **(C)** Relationship between HIV inoculum (x-axis; expressed as pg of HIV p24 capsid per cell) and the proportion ROS-producing cells (y left axis) discriminating within control, PI, and NPI/U astrocytes at 3 dpi. The percentage of HIV-infected cells is represented (y right axis; gray triangles). Data are presented as mean±SD. ^***^p < 0.0001 PI vs. Ctrl; $$$ *p* < 0.0001 PI vs. NPI, Student's *t*-test. **(D)** Relationship between geometric mean of Median Fluorescence Intensity (gMFI) of DCFDA assumed to be proportional to the concentration of hydrogen peroxide in control, PI, and NPI/U cells at 3 dpi, as previously shown in **(C)**. Data are presented as mean±SD. ^***^p < 0.0001 PI vs. Ctrl; $$$ *p* < 0.0001 PI vs. NPI, Student's *t*-test. **(E)** Histogram showing ROS generation at 3 dpi measured by FACS using DHE. The x axis represents the fluorescent signal intensity, and y axis the normalized cell number expressed as percentage of the maximum. Graphics are showing values obtained from 3 independent experiments. Data are presented as mean±SD. ^***^p < 0.0001 PI vs. Ctrl; $$$ *p* < 0.0001 PI vs. NPI, Student's *t*-test. **(F)** FACS contour plots from a representative experiment showing relative percentage of control, PI, and NPI/U astrocytes producing ROS (DCFDA) and Annexin-V co-staining. Graphics are showing values obtained from 3 independent experiments; small horizontal lines indicate the mean±SD. ^***^p < 0.0001 NPI vs. Ctrl; $$$ *p* < 0.0001 NPI vs. PI, Student's *t*-test.

We evaluated the relationship between HIV infection and generation of reactive oxygen species (ROS) in astrocytes. For this goal we measured by FACS two redox-sensitive fluorescent probes 3 days after HIV exposure: dihydroethidium (DHE) which is specific for superoxide (O${}_{2}^{-}$), and 2',7'-dichlorofluorescein-diacetate (DCFDA), that has higher specificity for H_2_O_2_. As expected, more than a 3-fold increase in the ratio of intracellular H_2_O_2_ production was observed in PI as compared to NPI/U, and more than a 16-fold increase as compared to control astrocytes (Figures 2A,B,D). Importantly, a pronounced shift in the geometric mean fluorescence intensity (gMFI) indicative of H_2_O_2_ content was observed in PI astrocytes (Figures 2B,D). Of note, H_2_O_2_ production increased proportionally to viral inoculum size (Figures 2C,D). Similarly, O${}_{2}^{-}$ was increased in PI astrocytes (Figure 2E). Hence, productive HIV infection of astrocytes causes significant increase in cellular ROS (Figures 2B,E).

Elevated ROS levels are related to DNA instability, Δψm dissipation and cell death. Among ROS, H_2_O_2_ is more stable and long-lived compared to O${}_{2}^{-}$, making it more suitable for cellular signaling as it may diffuse out of the mitochondria more easily due to its neutral ionic state. Our data indicated that despite the fact that the vast majority of HIV-PI astrocytes produce elevated H_2_O_2_ (84 ± 2%, Figure 2F), cell death is triggered only in a small proportion of them (15 ± 1%, Figure 2F). In contrast, almost 30% of the NPI/U dead cells did not produce H_2_O_2_ (28±8%, Figure 2F). These results indicate that HIV infection raises ROS in astrocyte irrespectively of its outcome as productive or abortive/null replication, but such ending may influence cellular fate, thus promoting survival for PI cells in contrast to cellular death for NPI/U cells.

### HIV-mediated mitochondrial damage occurs among bystander astrocytes

**Figure 3:**
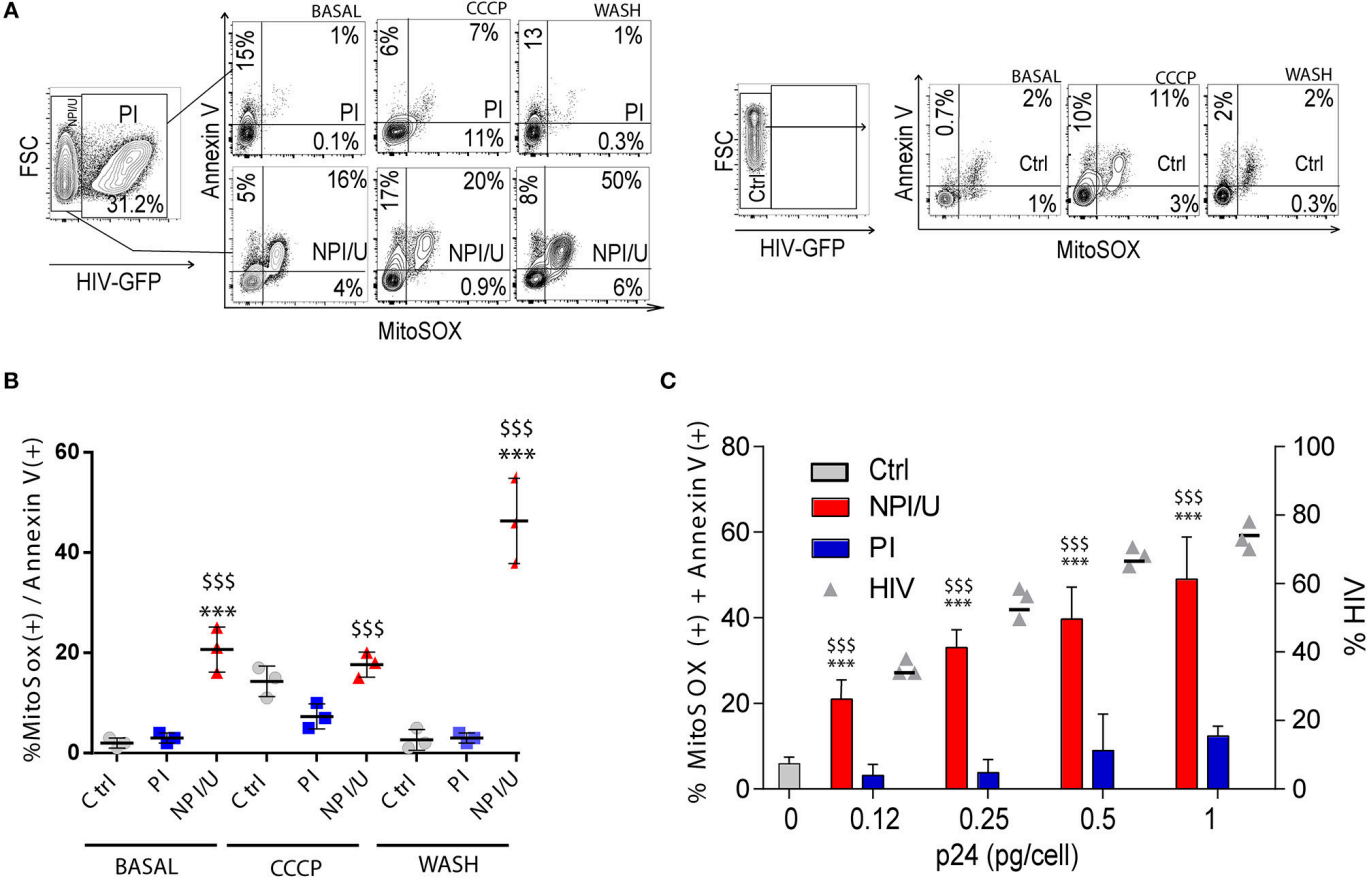
Analysis of mitochondrial damage (mROS generation, Δψm dissipation), cellular death, and inflammasome activation according to HIV-infection outcome. **(A)** Representative FACS contour plots obtained after control (mock) and HIV-exposed astrocytes were co-stained for mitochondrial ROS generation (MitoSox) and indications of cell death (Annexin V) at three distinguishable conditions: basal, after CCCP (50 μm) exposure, and after CCCP exposure followed by washing. **(B)** Graphic is showing values of co-stained mROS and annexin V astrocytes (at 3 dpi) measured by FACS from 3 independent experiments; small horizontal lines indicate the mean±SD. ^***^p < 0.0001 NPI vs. Ctrl; $$$ *p* < 0.0001 NPI vs. PI, Student's *t*-test. **(C)** Graphic is showing values of co-stained mROS and annexin V astrocytes exposed to different HIV inoculum (x axis, expressed as pg of HIV p24 capsid per cell), reaching different infection efficiency (y right axis, expressed as % of HIV infected cells, gray triangles). Values obtained from 3 independent experiments are represented; small horizontal lines indicate the mean±SD. ^***^p < 0.0001 NPI vs. Ctrl; $$$ *p* < 0.0001 NPI vs. PI, Student's *t*-test.

Another indication of mitochondrial damage is the increased mitochondrial ROS (mROS) production. For its selective measurement, we used a fluorogenic dye specific for superoxide detection in mitochondrial matrix of live cells (MitoSOX). Production of mROS was significantly higher in NPI/U than in PI and control cells (Figures 3A,B-“basal”). Furthermore, it was associated with annexin-V costaining (Figures 3A,B-“basal”). Taken together, these results show that a higher level of cell death occurs in NPI/U astrocytes than in PI-cells, in association with a more prominent mitochondrial damage (mROS production, Δψm dissipation; Figures 1D, 3A,B-“basal”). A plausible explanation for the co-occurrence of higher mROS levels, Δψm dissipation and death of bystander NPI/U cells is HIV-induced inflammasome activation.

We next examined whether mitochondrial damage is able to promote inflammasome activation in HIV-infected cultured cells treated CCCP (50 μm) (Figures 3A,B-“CCCP”), and observed a tightly increased mROS/annexin V costaining in control astrocytes (Figures 3A,B-“CCCP”) as well as the PI astrocytes (Figures 3A,B-“CCCP”). Such mitochondrial loss-of-function in control and PI cells induced after ΔΨm dissipation was reversible, as the removal of CCCP by washing fully restored the basal levels in both scenarios (Figures 3A,B-“wash”). Of note, CCCP treatment seemed to directly perturb mitochondrial physiology and increased mROS/annexin V levels in control and PI cells; however, before the washing a clearance mechanism is restoring mitochondrial damage (Figures 3A,B-“wash”).

NPI/U cells exhibited significantly increased mROS/annexin V levels after CCCP-treatment (Figures 3A,B-“CCCP”). Cell death and mROS production were sustained and augmented even after washing (Figures 3A,B-“wash”). Thus, these results support the model that inflammasome activators engage mitochondrial destabilization through specific stimulus in NPI/U cells, converging on the generation of particular signals associated with mitochondrial damage, finally leading to inflammasome activation. The mROS and annexin V levels in NPI/U cells were directly increased according to HIV inoculum size (Figure 3C) suggesting that inflammasome activation could be promoted from PI cells to NPI/U cells.

### HIV-infection promotes inflammasome activation as bystander effect

**Figure 4:**
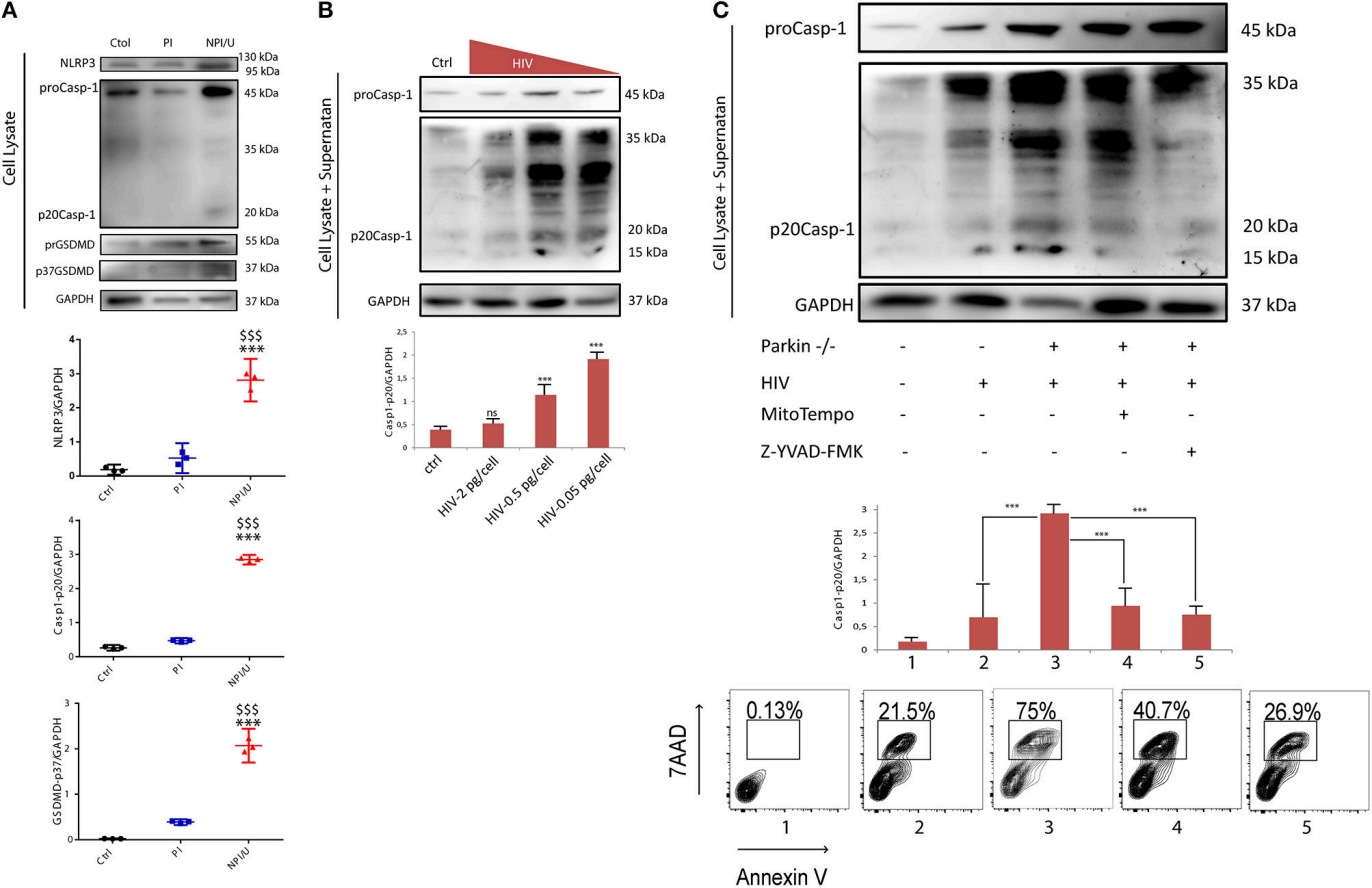
HIV infection in astrocytes and inflammasome activation as bystander effect. **(A)** Cell lysates extracted from astrocytes FACS sorted at 3 days post HIV infection were analyzed by western blot for NLRP3 expression, and cleavage of both caspase-1 (p20Casp-1) and Gasdermine D (p37GSDMD) in control, PI, and NPI/U. Separate graphics are showing normalized Caspase-1 (p20Casp-1) and GSDMD (p37GSDMD) values obtained from 3 independent experiments; small horizontal lines indicate the mean. Glyceraldehyde-3-phosphate dehydrogenase (GADPH) served as a loading control. ^***^p < 0.0001 NPI vs. Ctrl; $$$ *p* < ‘0.0001 NPI vs. PI, Student's *t*-test. **(B)** Level of proteolytically cleaved forms of caspase-1 (p20Casp-1) according to HIV inoculum used for *in vitro* infection. Combined supernatant and lysates were examined by Western blot for caspase-1 cleavage (casp-1p20). Separate graphic is showing p20Casp-1 values obtained from 3 independent experiments; small horizontal lines indicate the mean±SD. Glyceraldehyde-3-phosphate dehydrogenase (GADPH) served as a loading control. ***p < 0.0001 treatment vs. Ctrl, Student's *t*-test. **(C)** Cell lysates as well as cell culture supernatants were analyzed by western blot for cleavage of caspase-1. Control, HIV-infected astrocytes, and HIV-infected Parkin-knockdown (Parkin–/–) astrocytes are shown. Parkin-/- cells include 48 h treatment with a mitochondrial ROS scavenger (MitoTEMPO, Sigma-Aldrich) or, alternatively, with a caspase-1 inhibitor (Z-YVAD-FMK, Abcam). Separate graphic is showing Caspase-1 values obtained from 3 independent experiments; small horizontal lines indicate the mean ± SD. Glyceraldehyde-3-phosphate dehydrogenase (GADPH) served as a loading control. Representative FACS contour plots (obtained for each experimental condition) showing percentage of cell death (annexin-V^+^/7-AAD^+^).

It is widely accepted that NLRP3 inflammasome activity is positively regulated by mROS (15, 41, 42). To investigate the involvement of mROS production and Δψm dissipation of bystander NPI/U on inflammasome activation, the intracellular expression level of NLRP3, Caspase-1 and GSDMD (Gasdermin D) were evaluated after PI and NPI/U population separation by cell sorting at 3 dpi. The levels of NLRP3, active forms of Casp-1, and GSDMD -indicated by the appearance of its representative cleaved forms [Casp-1 (p20Casp-1) and GSDMD (p37GSDMD)] were identified in NPI/U but not among PI cells (Figure 4A). From these data we reasoned that mROS released by damaged mitochondria may promote NLRP3-inflammasome activation in NPI/U astrocytes. Given that only NPI/U cells promoted caspase-1 cleavage in HIV-infected cells, we tested whether increasing levels of HIV inoculums (0.05, 0.5, and 2.0 pg/cell of p24 antigen) decreased proportionally the level of active form of caspase-1 in HIV-infected astrocytes. As expected, lower viral inoculum promoted higher level of caspase-1 activation as a consequence of the high percentage of NPI/U cells in HIV-infected culture (Figure 4B).

Considering that mROS production and mitochondrial Δψm dissipation enhance inflammasome activation and caspase-1 cleavage, the role of Parkin as a critical protein for mitochondrial recycling was evaluated. To assess the relationships among Parkin, mROS, and inflammasome activation we evaluate the role of mitophagy inhibition during inflammasome activation using Parkin-depleted astrocytes (derived from shRNA mediated gene silencing). When Parkin was silenced a significantly higher level of Caspase-1 activation was observed (Figure 4C).

Finally, to test the hypothesis that mROS production from NPI/U astrocytes enhance NLRP3 inflammasome activation, a specific mitochondrial ROS scavenger (MitoTEMPO, Sigma-Aldrich) was added to HIV-cultured. Under this condition, the caspase-1 activation was significantly lower and, consequently cell death was diminished. By inhibiting caspase-1 activity (using Z-YVAD-FMK, Abcam), in spite of increased expression of pro-caspase-1 (45 kDa), a negligible level of mature caspase-1 (20 kDa) was observed (Figure 4B). It was correlated with significant decreased in cell-death observed by FACS (Figure 4C).

### HIV infection promotes mitochondrial fission in PI and NPI/U astrocytes

**Figure 5:**
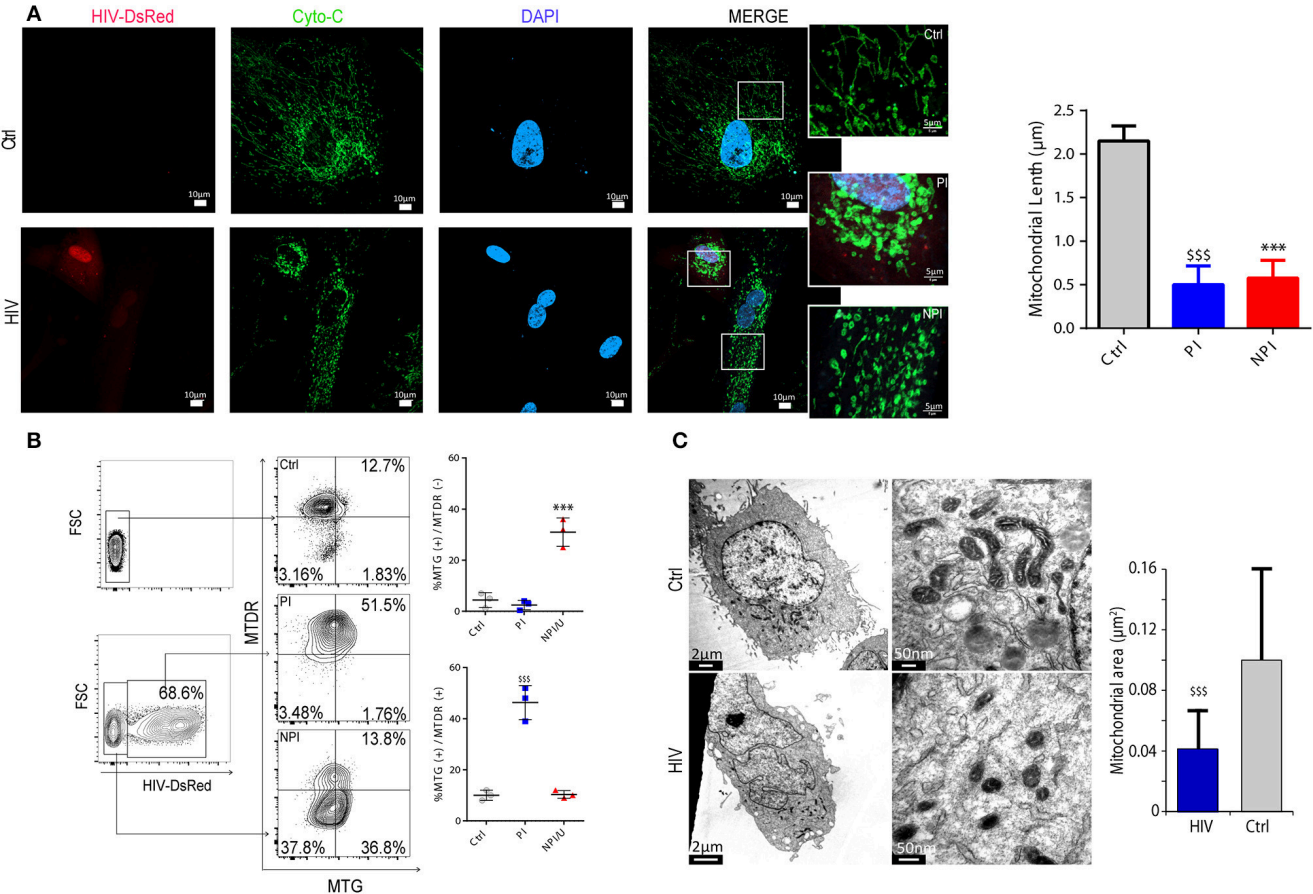
HIV infection promotes mitochondrial fission in astrocytes. **(A)** Confocal fluorescence microscopy showing mitochondrial morphology (Cytochrome C Alexa Fluor® 488) in HIV (expressing DsRed fluorescence) infected (PI), non-infected (NPI/U), and unexposed control astrocytes. Mitochondria exhibiting a filamentous pattern are observed in unexposed control cells, but fragmented mitochondria in HIV-infected (PI) and non-infected (NPI/U) astrocytes. Inserted figures are magnification of selected regions in order to better visualize mitochondrial morphological differences. Quantification of mitochondrial length in PI, NPI/U and control astrocytes is represented; values were obtained from a representative experiment performed in triplicate. ^***^p < 0.0001 NPI vs. Ctrl; $$$ *p* < 0.0001 PI vs. Ctrl, Student's *t*-test. **(B)** Mitochondrial depolarization (MitoTracker™ Deep Red, MTDR) and mitochondrial swelling (MitoTracker™ Green, MTG) occurrence for control, PI (DsRed+), and NPI/U astrocytes is shown. Mitochondrial swelling was observed for those gated cells stained more brightly with MTG. Mitochondrial depolarization occurs in cells stained less brightly with MTDR. Control and PI cells preserve their mitochondrial morphology and ΔΨm in contrast with NPI/U cells. **(C)** Transmission electron microscopy (TEM) ultrastructure of PI and control unexposed cells showing mitochondrial morphology. At 3 days post infection astrocytes were sorted by FACS previously to TEM in order to visualize HIV-infected enriched population cells. In the zoomed images, mitochondria from PI cells depict fragmentation resulting in a significant reduction in organelle area. The bar graph is showing a representative experiment performed in triplicate. $$$ *p* < 0.0001 PI vs. Ctrl, Student's *t*-test.

Mitochondria can be the source of ROS but also the target of oxidative damage or inflammasome activation during cellular stress. Using fluorescence confocal microscopy on HIV-exposed cells at 3 dpi stained with primary anti-Cytochrome C. Significant morphological changes in both NPI and PI astrocytes were observed (Figure 5A). While the mitochondrial network in control cells appears largely tubular, both HIV-exposed cells (PI and NPI/U astrocytes) displayed fragmented morphology, appearing rounded with reduced length (Figures 5A–C). Such fragmentation may lead to two outcomes (28) depending on the scenario: mitochondrial clearance by mitophagy, or alternatively, inflammasome activation (19, 43). Supporting this assumption, the MTG staining was increased in both NPI/U and PI cells (Figure 5B), but membrane potential dissipation and increased mROS production was manifested only in NPI/U astrocytes (Figure 5B). As shown in Figure 5B, such mitochondrial fission and damage could be related to caspase-1-mediated cleavage (Figure 4A) of proteins related to mitochondrial dynamics after inflammasome activation, but further research is deserved to elucidate the details of this phenomenon (28).

After simultaneous FACS analysis with MTDR (MitoTracker™ Deep Red as mitochondrial dye that is taken up in a ΔΨm-dependent manner) and MTG, NPI/U cells showed simultaneous increase of mitochondrial swelling and mitochondria membrane depolarization (MTG^+^/MTDR^−^; Figure 5B), supporting the hypothesis that the activation of Caspase-1 reinforces mitochondrial damage and fragmentation. However, PI astrocytes depicted increased mitochondrial fragmentation but preserved ΔΨm (MTG^+^/MTDR^+^; Figure 5B). Mitochondrial fragmentation in PI cells may be related to increased ROS levels as reported by several authors (31, 44). However, the mitochondrial fission is not associated with the loss of membrane potential in PI astrocytes. These morphological differences were confirmed by studies using transmission electron microscopy that also enabled us to determine the mitochondrial ultrastructure. As shown in Figure 5C, mitochondrial fragmentation was significantly higher in HIV-PI astrocytes than control cells, and it was accompanied by a pronounced reduction in mitochondrial area. In conclusion, after HIV exposure, loss of mitochondrial membrane potential and mitochondrial fragmentation was observed among NPI/U astrocytes. The same pattern of mitochondrial fragmentation was evident in PI astrocytes without ΔΨm dissipation. These findings support the model that inflammasome activators in NPI cells engage mitochondrial destabilization via stimulus-specific mechanisms leading to inflammasome activation and Caspase-1–dependent increase of mitochondrial damage (e.g., increased mROS production by CCCP; Figure 3A). However, in the PI astrocytes no activation of the inflammasome is registered (Figure 4A), and apparently the alteration in the mitochondrial morphology (Figures 5A,B) may be indicative for a rescue strategy such as mitophagy.

### HIV-mediated mitochondrial damage involves cell-to-cell interaction

**Figure 6:**
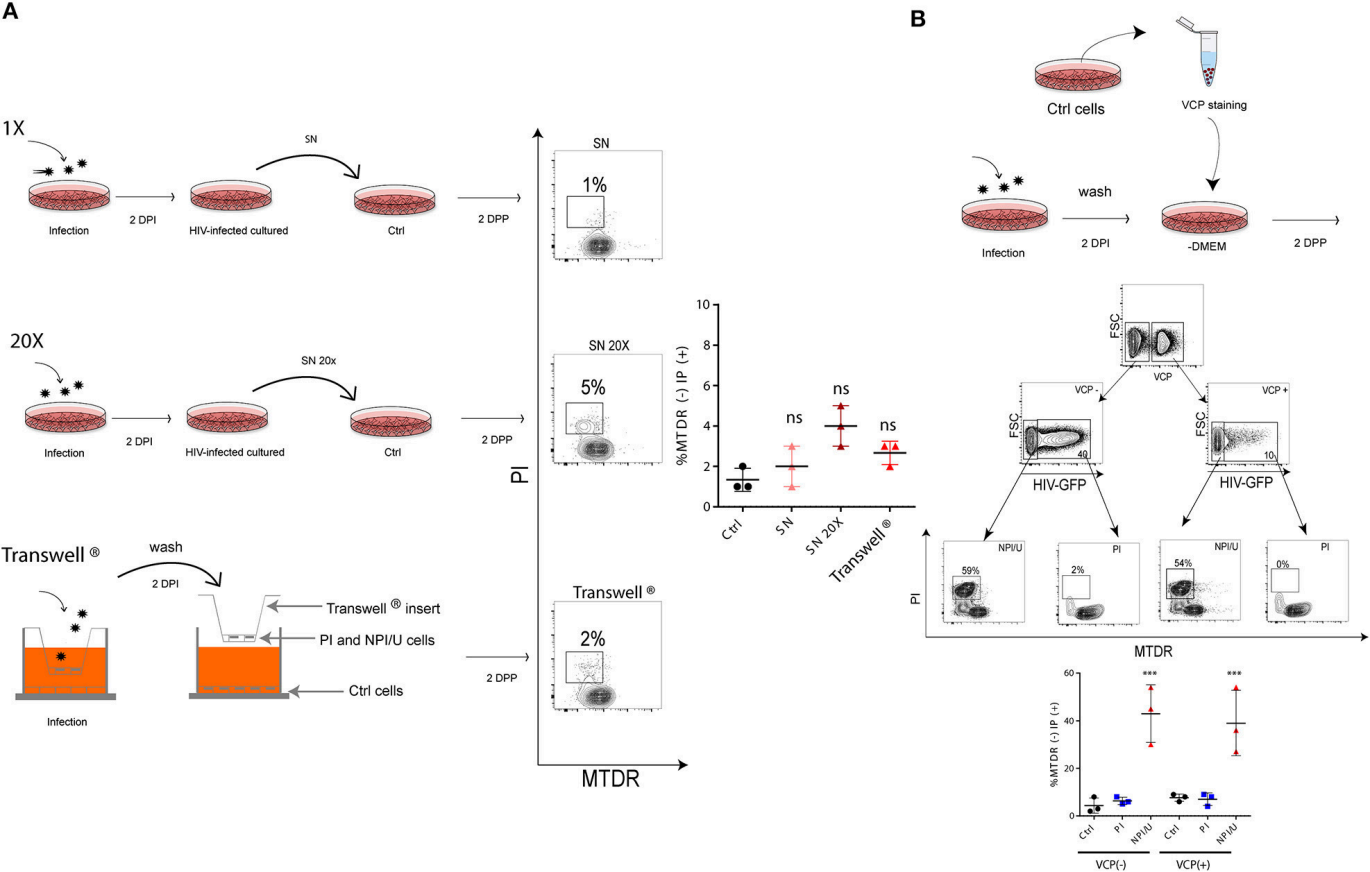
Cell-to-cell contact is necessary to promote bystander mitochondrial damage among HIV- exposed astrocytes. **(A)** FACS contour plots from representative experiments. Each one is showing cell membrane permeability (IP) and mitochondrial membrane depolarization (MTDR) in targeted astrocytes challenged during 2 days with 1X (top), 20X (middle) conditioned medium (astrocytes culture supernatant obtained after 2 dpi with HIV), and (bottom) targeted astrocytes co-cultivated with infected cells cultured on the top chamber (using Transwell® system, pore a size 0.6 μm). The graph is showing values obtained from 3 independent experiments; small horizontal lines indicate the mean. **(B)** FACS contour plots from a representative experiment. Targeted astrocytes were stained with Violet Cell Proliferation Dye (VCP) and then cocultured with a 2 dpi HIV-infected culture. Two-days after cell-to-cell contact, “bystander” astrocytes (VCP stained cells) were differentiated, and HIV-productive infection was measured (GFP) for each cell population using FACS. For each differentiated astrocyte population both mitochondrial damage (MTDR) and cell membrane permeability (IP) were measured. Graphic is showing values obtained from 3 independent experiments; small horizontal lines indicate the mean. ***p < 0.0001 NPI vs. Ctrl, Student's *t*-test.

It is uncertain whether inflammasome activation and mitochondrial damage occur after direct cell-to-cell contact among HIV-exposed astrocytes or, alternatively, can be fully supported by free virus (and soluble viral proteins) released in supernatants after HIV replication. Using cell-free supernatants from HIV-infected astrocytes, negligible levels of cell membrane permeability alterations and mitochondrial damage were observed (Figure 6A). Similarly, despite challenging the cells with a 20-fold concentrated on viral inoculum (p24 = 1 mg/ml) both parameters did not show substantial changes (Figure 6A). Moreover, similar findings were obtained using a transwell culture system that allowed separating physically non-exposed control and HIV-infected astrocytes by a transwell insert with 0.6 μm pores (Figure 6A).

The cell-to-cell contact was assessed using CellTrace™ Violet Cell Proliferation (VCP) assay. VCPneg-HIV exposed cells (including PI and NPI/U cells) were prompted to contact with VCPpos-uninfected cells by co-culture over 2 days. As a result, two VCPpos and two VCPneg-cell populations were distinguishable according to HIV-GFP expression by flow cytometry. Alterations in both mitochondrial (i.e., ΔΨm dissipation using MTDR dye) and cellular membrane integrity (by staining with propidium iodide) were observed in NPI/U (GFP negative) populations from VCP-positive and VCP-negative cells. However, these abnormalities among GFP-positive cells originally infected by HIV (VCP-negative) and those “innocent” cells infected after cell-to-cell contact (VCP-positive) were not observed. Collectively, these observations demonstrate that bystander effects on astrocytes including mitochondrial damage and cellular membrane alterations observed after HIV exposure are mediated by cell-to-cell contact between PI–NPI/U cells (Figure 6B).

### Autophagy is induced on HIV-productively infected astrocytes

**Figure 7:**
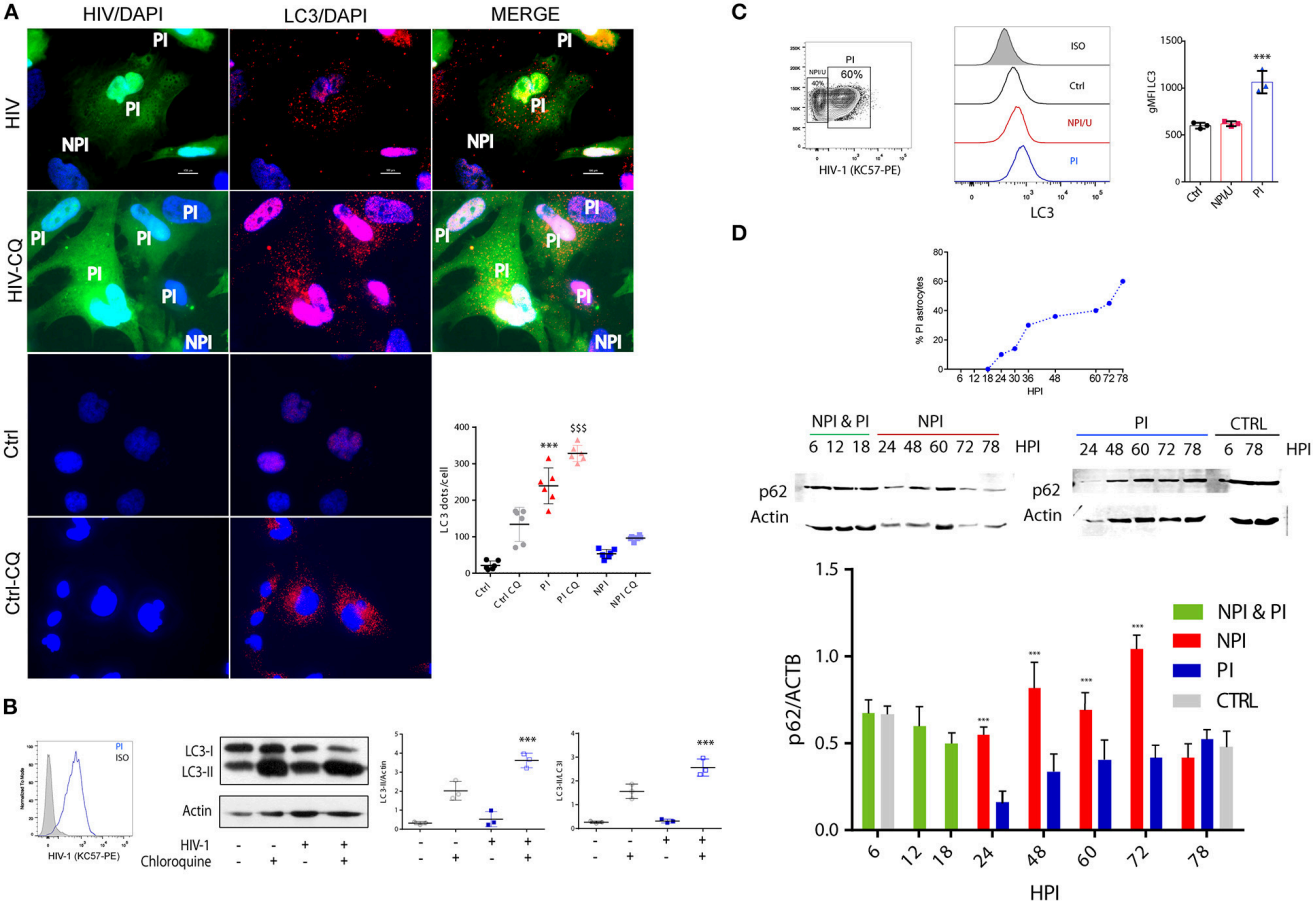
Autophagy is differently promoted between PI and NPI/U astrocytes. **(A)** Fluorescence microscopy is showing LC3 dot pattern in HIV-infected cells. At 3 day post-infection human astrocytes were immunostained with anti-LC3 (AF 647). Alternatively, cells were treated with chloroquine (CQ, 50 μm) for 2 h in full medium before fixation for autophagy flux evaluation. The number of LC3 punctae per cell was quantified; 50 cells were analyzed per assay. Bars graphic is showing mean values (±SD) from five independent experiments. ^***^p < 0.0001 PI vs. Ctrl; $$$ *p* < 0.0001 PI-CQ vs. Ctrl-CQ, Student's *t*-test. **(B)** Histogram overlays are showing the amount of intracellular p24 expression (measured by anti-p24 antibody KC57-PE) in HIV-infected astrocytes (PI). Western blot is showing the LC3 processing (conversion from LC3-I to LC3-II form) in PI and control cells. Besides, results showing autophagy flux evaluation are depicted; for this goal astrocytes were treated with CQ (50 μm) for 2 h. β-actin served as a loading control. Pooled data from 3 independent experiments are shown for both LC3II/actin and LC3II/LC3I ratios. ^***^p < 0.0001 PI-CQ vs. Ctrl-CQ; Student's *t*-test. **(C)** A representative counter plot is showing HIV-infection rate (depicted as intracellular p24 expression) in cultured astrocytes. Cellular extracts obtained after saponin treatment (as part of the protocol for p24 intracellular staining) were also used for LC3-II fluorescence quantification by FACS and expressed as median fluorescence intensity (gMFI). Histogram overlays and gMFI values of intracellular LC3 on PI, NPI/U and control cells are shown. Pooled data from 3 independent experiments is shown. ^***^p < 0.0001 PI vs. Ctrl; Student's *t*-test. **(D)** Graphic is showing the dynamics of GFP+ cells (PI) sorted by FACS according to infection progress. At 6, 12 and 18 h post-infection (hpi) PI/NPI cells could not be sorted and NPI & PI cells are in the same lysate. Western blot is showing p62 protein expression for PI and NPI/U astrocytes. β-actin served as a loading control. Bars graphic represents pooled data from 3 independent experiments. ^***^p < 0.0001 NPI vs. Ctrl, Student's *t*-test.

Because autophagy is an important response to cellular stress, we next sought to determine whether HIV infection alters autophagy in astrocytes. To this aim, 72 h post HIV exposure, astrocytes were fixed and labeled with anti-LC3 as a marker of autophagosomes (30). Confocal fluorescence microscopy imaging of control cells revealed a diffuse pattern of LC3 fluorescence, suggesting low to negligible levels of basal autophagy (Figure 7A). In contrast, HIV-PI cells exhibited significant accumulation of LC3-positive punctate structures, suggesting recruitment of LC3 to autophagosomes at 3 dpi (Figure 7A). However, NPI/U cells showed a diffuse LC3 staining pattern similar to control cells, advising irrelevant autophagy activation. To confirm that autophagy is induced by HIV, we quantified the number of LC3 dots per cells after chloroquine (CQ) treatment to inhibit lysosomes acidification thus effectively blocking the last step of autophagy flux (30) (Figure 7A). Endogenous LC3 expression was visualized by fluorescence microscopy as a diffuse cytoplasmic pool in CQ-treated NPI/U and control cells, and as punctate structures in CQ-treated PI cells.

To confirm the activation of autophagy in HIV productive infected astrocytes, we analyzed the conversion of the cytosolic form of LC3 (LC3-I) to its LC3-phosphatidylethanolamine conjugate (LC3-II) that is recruited to autophagosomal membranes, by western blotting (30). Under this condition, a significant increase in LC3-II/LC3-I and LC3II/actin ratio was observed in HIV-PI cells as compared to control cells (Figure 7B). To determine whether the observed increase in autophagosome number in HIV-PI cells resulted from an increase in autophagic flux, we treated HIV-PI astrocytes with CQ (50 μm) for 2 h. CQ-treated HIV-PI cells showed a significant increase in LC3-II abundance (Figure 7B), providing conclusive evidence for an increase in autophagy flux in HIV-productively infected cells.

Additionally, autophagy activation patterns in different cell population according to HIV-infection status (NPI/U, PI, and control) were performed by flow cytometry analysis. Soluble LC3-I can be depleted from cell by a brief saponin extraction, so that the total fluorescence represents only LC3-II (30). As depicted in Figure 7C, histogram plots of cell populations were employed in order to quantify autophagy rate. Changes in LC3 mean fluorescence intensity (gMFI-LC3) in PI cells were significantly higher than in control and NPI/U cells, but no differences were observed between NPI/U and control cells (Figure 7C).

Furthermore, autophagic flux was analyzed among sorted HIV-infected cells. For this goal, p62 quantification was separately carried out in both PI and NPI/U cell populations by western blotting. PI astrocytes exhibited an inverse correlation between LC3-II increase and SQSTM1/p62 decrease. In contrast, a marked increase in p62 amount in NPI/U astrocytes was evident at 24, 48, 60, and 72 h post infection (Figure 7D). In conclusion, the analysis of autophagic activity unraveled two opposite behaviors between HIV-exposed subpopulations in that autophagic flux was augmented in PI cells but inhibited in NPI/U astrocytes (Figure 7D).

### Cell death resistance in HIV-productively infected cells is facilitated by mitochondrial fission and mitophagy

Depending on HIV infection outcome, two distinct cellular scenarios were characterized. Human astrocytes that were productively infected exhibited lower mitochondrial damage and cell death but higher autophagy flux than NPI/U ones. The latter strategy may allow to surpass mitochondrial dysfunction and consequently to attenuate intracellular accumulation of mROS, inflammasome activation, and cell death. In consequence, we wondered whether damaged mitochondria undergoing fragmentation in PI astrocytes segregate from healthy pool to be recycled by mitochondria-selective autophagy (mitophagy). We hypothesized that HIV may specifically stimulate mitophagy as a protective response to mitochondrial dysfunction and increased ROS production.

**Figure 8:**
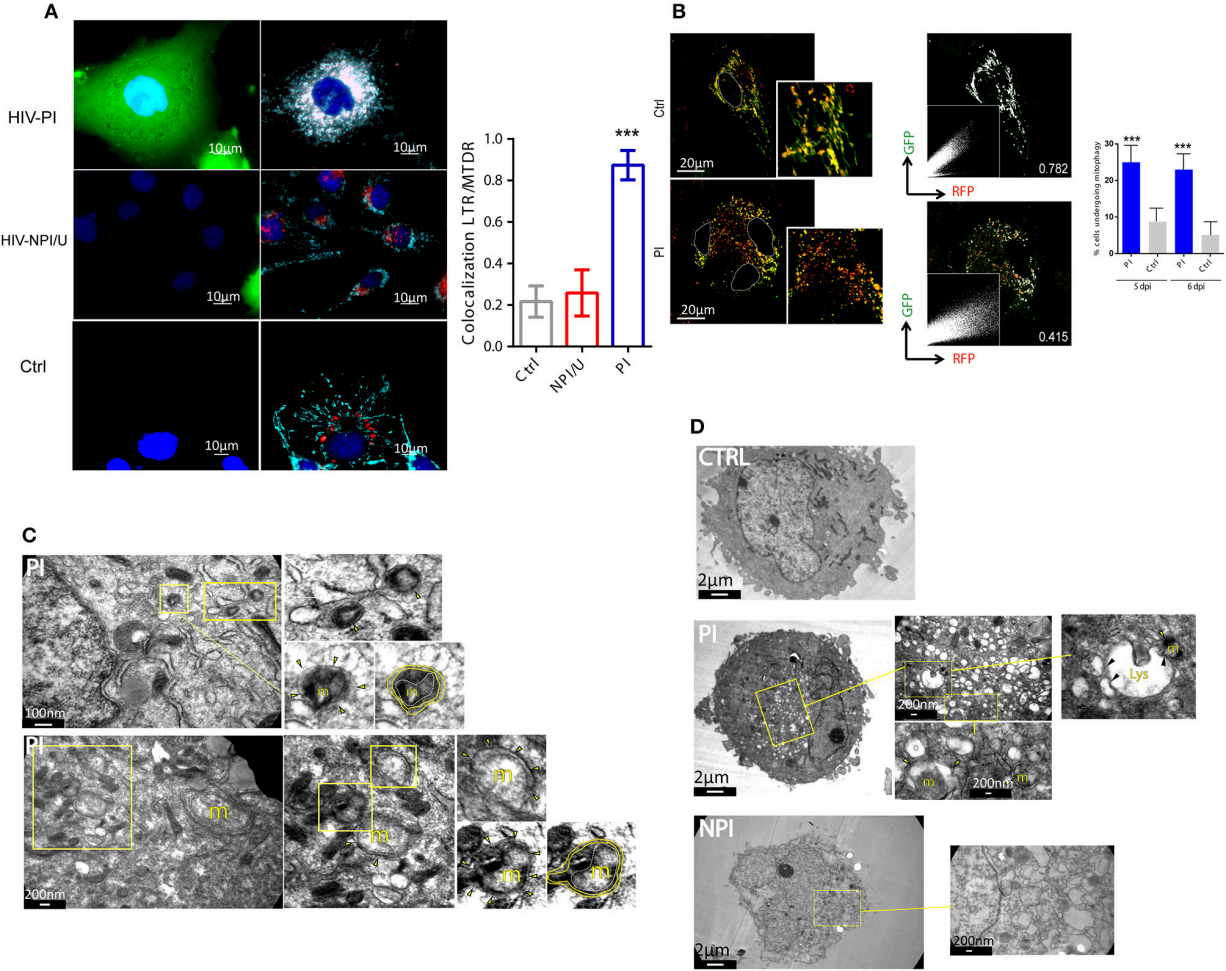
Mitophagy is promoted in HIV-productively infected cells. **(A)** Fluorescence microscopy showing colocalization of lysosomes stained with LysoTracker™ Green (LTG, 100 nm), and mitochondria stained with MitoTracker™ Deep Red (MTDR, 100 nm) in HIV-exposed astrocytes (PI and NPI/U) and control cells at 3 dpi. A significant colocalization and fragmentation is observed in PI cells. Bar graph is showing means (±SD) from three independent experiments. ^***^p < 0.0001 PI vs. Ctrl; Student's *t*-test. **(B)** Differential expression of pAT016-mRFP-EGFP among astrocytes transfected with pAT016 reporter plasmid and exposed to HIV. Filamentous yellow (high channels colocalization, GFP^+^/RFP^+^) healthy mitochondria are observed in control cells. In contrast, fragmented mitochondria with increase in red dots (low channels colocalization, GFP^−^/RFP^+^), indicative of mitophagy, are observed in HIV infected astrocytes. These observations are supported by the overlapped white pseudo-color for colocalization areas (right panels) and the corresponding Pearson correlation for red and green channels (Pearson correlation coefficients are depicted on image). Right inserts represent red and green channel dot plots. Plots showing increased mitophagy percentage in HIV infected cells versus control, after 5 and 6 days post infection. Bar graph is showing pooled data from 3 independent experiments. ^***^p < 0.0001 PI vs. Ctrl, Student's *t*-test. **(C)** Representative transmission electron microscopy (TEMs) of HIV infected astrocytes showing examples of structures compatible with mitophagy process (m: mitochondria; arrowhead: indicates autophagosomes; yellow line: autophagosomal membranes; white line: outer mitochondrial membrane). **(D)** Representative TEMs of HIV infected astrocytes showing high number of autophagic vesicles. Zoomed middle panels show examples of mitophagy. Left zoomed image shows a mitochondria-containing autophagosome (arrowhead) approaching the lysosome (m: mitochondria; arrowhead: indicates mitochondria-containing autophagosomes; Lys: lysosome; black arrowhead: vesicle-lysosome fusion points).

To elucidate whether mitophagy was selectively activated in HIV-PI cells, the co-localization of mitochondrial and autophagosomal/lysosomal markers was evaluated. HIV-PI cells showed colocalization and perinuclear clustering of MitoTracker™ Deep Red (MTDR) and LysoTracker™ Red (LTR). In contrast, in NPI/U and unexposed control cells, colocalization was not observed and the organelle distribution appeared normal (Figure 8A).

The evaluation of the final step of mitophagy (fusion of mitophagosomes with lysosomes) using pAT016 among HIV-unexposed control cells showed abundant filamentous organelles exhibiting yellow signal as product of colocalized red and green fluorescence as indicative of stable intact mitochondria. In contrast, HIV-PI cells showed large numbers of fragmented mitochondria with predominantly red fluorescence, suggesting a robust increase in mitophagy (Figure 8B). To further validate such increase in mitophagy on HIV-PI astrocytes, we examined these cells using transmission electron microscopy. As depicted in Figures 8C,D, both mitophagosomes and mitophagosomes fused with lysosomes were observed in HIV-PI but not in HIV-unexposed control cells.

**Figure 9:**
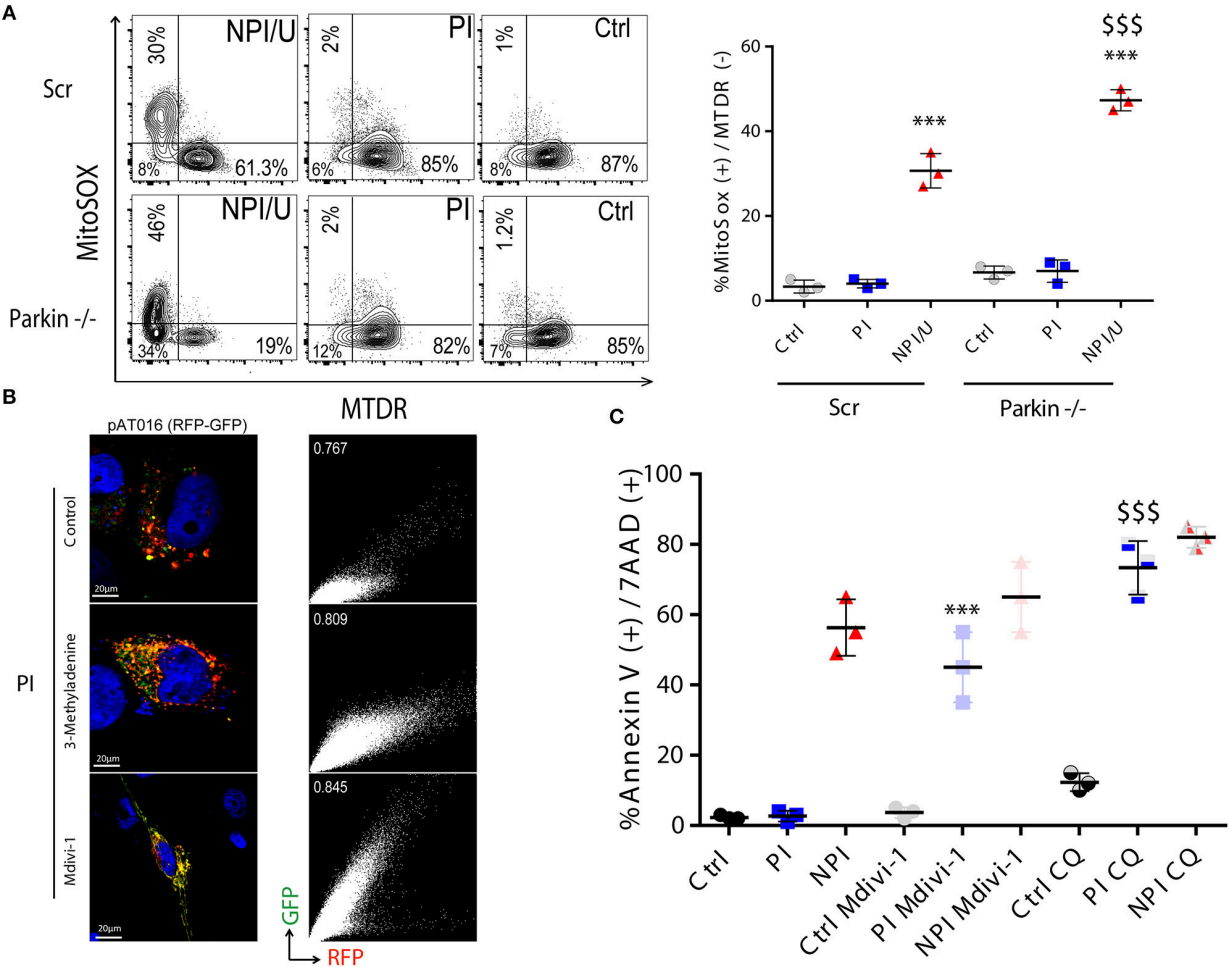
Mitophagy in PI astrocytes is associated with cell death resistance. **(A)** FACS contour plots from a representative experiment showing mitochondrial membrane potential dissipation (MitoTracker™ Deep Red, MTDR) and mitochondrial ROS production (MitoSox™) in control, PI, and NPI/U astrocytes at 3 dpi. Two distinguishable conditions were studied according to Parkin expression such as control cells that were transduced with scramble (Scr), and Parkin knockdown (-/-) astrocytes. Graphics are showing values obtained from 3 independent experiments; small horizontal lines indicate the mean±SD. ^***^p < 0.0001 NPI vs. Ctrl; $$$ *p* < 0.005 Parkin-/-(NPI) vs. Scr (NPI), Student's *t*-test. **(B)** Differential expression of pAT016-mRFP-EGFP among PI-astrocytes transfected with pAT016 reporter plasmid and exposed to HIV (as Figure 7B). Alternatively, cells were treated with the early autophagy inhibitor 3-methyladenine (5 mm) or a mitochondrial fission inhibitor (Mdivi-1, 50 μm). Right inserts depict Pearson correlation coefficient for red and green channels colocalization. **(C)** Increased cell death is observed among PI-astrocytes after autophagy/mitophagy inhibition. Graphic is showing values of cells death (annexin V^+^/7-AAD^+^) in PI and control cells, with and without autophagy inhibitor chloroquine (50 μm), or a mitochondrial fission inhibitor, Mdivi-1 (50 μm) treatment. Represented data were obtained from 3 independent experiments; small horizontal lines indicate the mean. ^***^p < 0.0001 PI-Mdivi-1 vs. Ctrl-Mdivi-1; $$$ *p* < 0.0001 PI-CQ vs. Ctrl-CQ, Student's *t*-test.

According to Parkin-dependence, at least two basic molecular mechanisms are described for facilitating mitophagy which may be relevant in a given cellular context (45–47). To evaluate its occurrence we determined the relevance of mitophagy inhibition during inflammasome activation using Parkin-depleted astrocytes (derived from shRNA mediated gene silencing). These cells showed higher mitochondrial damage (mROS production and mitochondrial membrane depolarization) when Parkin-dependent mitophagy is deteriorated (Figure 9A). In contrast, control and PI cells did not exhibit neither loss of ΔΨm nor increased mROS production in both Parkin-/- condition and negative control (using a scrambled version of shRNA; Figure 9A).

We next aimed to elucidate whether mitochondrial fission occurs as consequence of productive HIV-infection that would propitiate posterior mitophagy. After HIV-PI astrocytes and unexposed control cells expressing pAT016 were treated with Mdivi-1 (inhibitor of Drp-1 required for mitochondrial fission), or with 3-methyladenine (3-MA, an inhibitor of early steps during autophagy induction), a significant reduction in mitophagy was observed (Figure 9B). Taken together, these data suggest that HIV-productive infection induces mitochondrial fission followed by mitophagy, as a plausible mechanism to recycle damaged mitochondria

Interestingly, in spite of high ROS production, productive HIV PI astrocytes exhibited increased resistance to cell death involving autophagy and mitochondrial fission. When such processes were blocked (with CQ, 3-MA, and Mdivi-1), cell death resistance was abolished (Figure 9C).

**Figure 10:**
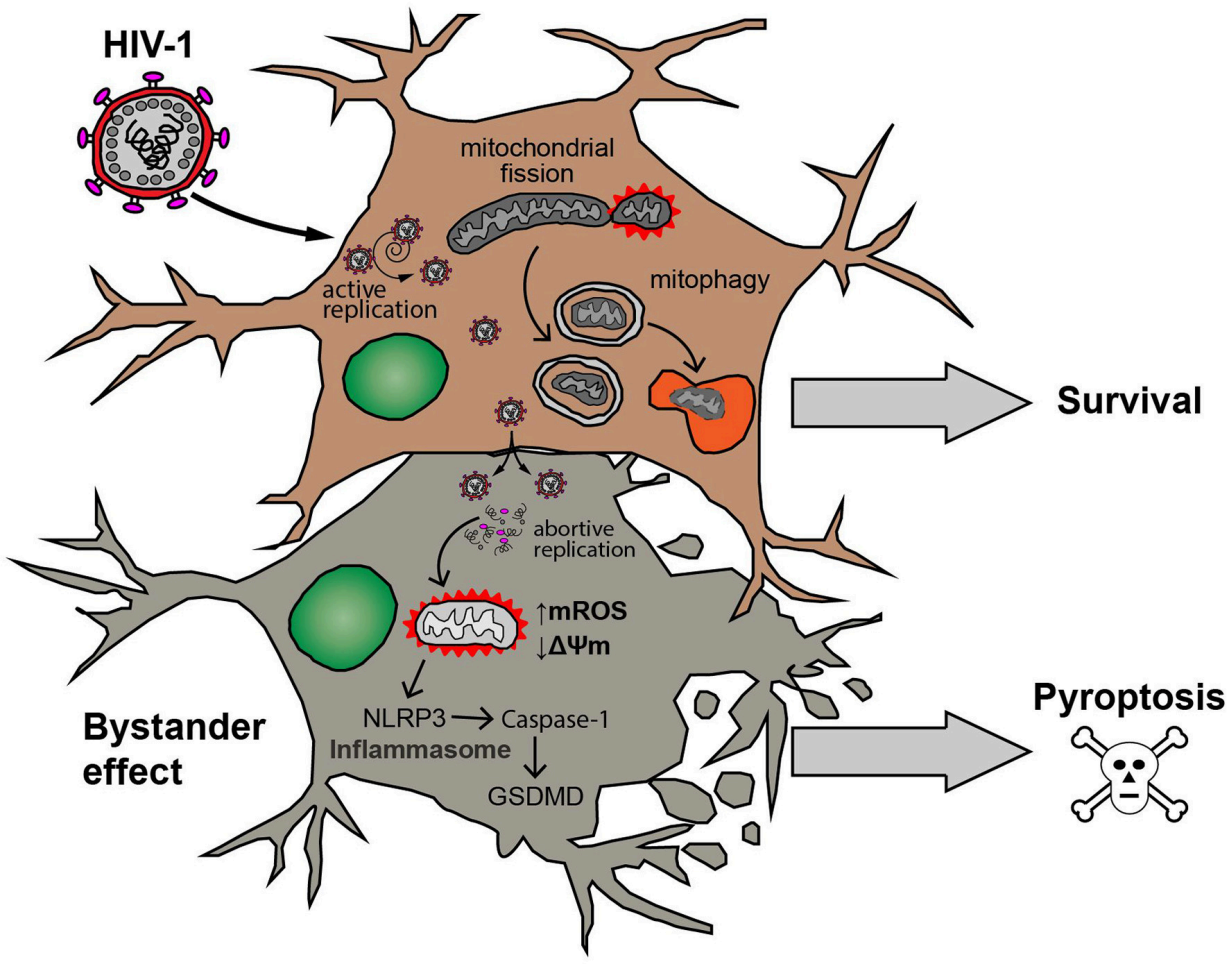
Model of HIV-1 infection outcome in human astrocytes. Astrocyte productively infected with HIV-1 (upper cell) is able to attenuate both mitochondrial ROS production (↑mROS) and mitochondrial membrane potential dissipation (↓*ΔΨ*m). Such mitochondrial injury is counteracted by increasing of mitochondrial fission processes and elimination of damaged mitochondria by mitophagy resulting in survival of the cell. On the other hand, a bystander effect occurs after cell-to-cell contact with HIV-productively infected astrocytes. In these cells (schematized in lower example cell), abortive viral replication induces mitochondrial oxidative stress, inflammasome activation (NLRP3-Caspase-1-GSDMD pathway) and eventually the cell death by pyroptosis.

Collectively, these results propose that after HIV exposure, astrocytes depict a differential involvement of inflammasome-triggered cell death according to infection outcome. Thus, NPI/U but not PI cells showed exacerbated inflammasome activation, with consequent mitochondrial damage, and cell death. Parkin-dependent mitophagy among NPI/U but not PI cells may ameliorate cell death. Among PI-astrocytes mitophagy is triggered in a Parkin-independent way (Figure 10).

## Discussion

The pathways causing cell death in HIV-infected CD4^+^ T cells are still not completely elucidated although apoptosis and pyroptosis have been proposed as alternative pivotal mechanisms depending on the outcome of infection (48–51).

Astrocytes may represent an HIV cellular reservoir within the CNS because they are abundant and display a high frequency of infection (2, 52). The HIV infection outcome on these cells is less clear, although a previous report has shown that they may survive or undergo cell death, dependent on viral replication or innocent bystander effects including mitochondrial dysregulation, respectively (3). Mitochondrial damage and fragmentation by fission may be triggered, among others, by virus gene expression, ROS elevation, or mitochondrial membrane potential dissipation (Δψm) (34, 53). At this point, effective mitochondrial quality control is critical. Such damaged or fragmented mitochondria may undergo fusion under certain conditions. Otherwise, they can be subjected to selective mitophagy thus favoring cellular viability, or alternatively trigger pyroptosis (involving NLRP3 inflammasome activation, caspase-1 cleavage, and GSDMD cleavage) (28, 54). As recently reported, such opposed mechanisms may interact reciprocally in viral infection and sepsis (19, 55, 56).

In this study, we demonstrate that human astrocytes exposed to HIV, either producing viral progeny or not, exhibited elevated cytoplasmic ROS (including H_2_O_2_ and O${}_{2}^{-}$) and mitochondrial fragmentation. Importantly, other intimately related consequences depend on the viral infection outcome. In particular, we observed that a physiological elevation of ROS production during HIV productive replication was directly related to the magnitude of efficiency of replication, but were accompanied paradoxically by an unaltered Δψm and an attenuated production of mROS. These preserved mitochondrial parameters may be reached by selective fragmentation and selective autophagy of damaged mitochondria, which could be explained by their rapid engulfment during mitophagy (57).

Such productively infected astrocytes were able to block the NLRP3 inflammasome activation by inducing mitophagy, thus facilitating the removal of damaged mitochondria. Moreover, we also found that productively infected astrocytes promote autophagy, inducing autophagosome formation as evidenced by the presence of LC3 positive punctate structures as well as the autophagic flux as shown by processing of LC3-I to LC3-II. The latter was reinforced by lower levels of p62 which also acts as an essential mediator of mitophagic elimination of mitochondria that were damaged by active caspase-1 (58). Furthermore, after HIV infection of Parkin-deficient astrocytes, an enhancement of Δψm dissipation and mROS production in non-productively/uninfected cells were observed, suggesting that impaired mitophagy can be a source of danger signals that act directly as NLRP3 inflammasome activators as a bystander effect. Besides, mitochondria of neighboring non-productively infected and/or uninfected cells are target of ROS, which trigger cell death. These cells evidenced mitochondrial damage as a bystander effect depicted by exacerbated mROS production and dissipation of Δψm.

In agreement with the hypothesis that mitophagy functions as a quality control mechanism that influences mitochondrial turnover, it has been proposed that mitochondrial fragmentation contributes to the segregation of less active mitochondria that are later targeted by the mitophagy machinery (45, 57). Here, we demonstrated that blocking fission and mitophagy restored the sensitivity of HIV-productively infected astrocytes to cell death, suggesting that mitochondrial fission and mitophagy are required for cell death resistance, thus favoring cell survival in HIV-infected astrocytes.

Considering that HIV infection of astrocytes may be mediated by cell-to-cell contact (4), this strategy could also influence bystander death on neighboring abortively infected/uninfected astrocytes. In this sense, we have advised that innocent bystander effects (mitochondrial damage and plasmatic membrane alteration) depend on cell-to-cell contact between these cells and productively infected ones. In contrast, progeny free virus was not able to trigger such phenomena. Such contagious cell death may be mediated by DAMP- (i.e., ROS, mitochondrial DNA) and/or PAMP (viral proteins, viral RNA or DNA) mediated signaling but further investigation of the underlying pathways is deserved.

Taken together, our data provide the first evidence that productive HIV replication on astrocytes triggers a sequential cascade of events involving the regulation of mitochondrial dynamics and degradation through the activation of mitochondrial fission followed by mitophagy. These findings provide novel insights into the regulation of mitochondrial dynamics and cell survival during the HIV infection cycle, and suggest that inhibiting mitochondrial fission or mitophagy may constitute potential therapeutic strategies aimed at reducing or eliminating HIV reservoirs in the brain.

## Author contributions

DO, DG, MV, and JQ contributed to the conception and design of this study. DO, DG, MV, and JQ were responsible for the acquisition, analysis and interpretation of data, and to the writing of the manuscript. DO, DG, and AT focused on bioinformatics analysis and data interpretation. DO, JU, and DG contributed to cell biology and molecular biology experiments. AT was responsible for quantitative image analysis and provided expertise on reporter gene- based mitophagy assays.

### Conflict of interest statement

The authors declare that the research was conducted in the absence of any commercial or financial relationships that could be construed as a potential conflict of interest.

## Abbreviations

3-MA: 3-methyladenine
ATP: Adenosine triphosphate
BNIP3: BCL2 interacting protein 3
CCCP: carbonyl cyanide m-chlorophenyl hydrazine
CD4: cluster of differentiation 4
CNS: central nervous system
CQ: chloroquine
CXCR-4: C-X-C chemokine receptor type 4
DCFDA: dichlorofluorescin diacetate
DNA: deoxyribonucleic acid
dpi: Days post infection
FACS: fluorescence-activated cell sorting
FUNDC1: Fun14 domain containing 1
GFP: green fluorescent protein
HIV: human immunodeficiency virus
LC3: microtubule-associated protein 1 light chain 3
Mdivi-1: mitochondrial division inhibitor-1
MitoSOX: mitochondrial superoxide indicator
mRNA: messenger RNA
mROS: mitochondrial reactive oxygen species
MTDR: mito tracker deep red
MTG: mito tracker green
NPI/U: productively infected and/or uninfected
OMM: outer mitochondrial membrane
PE: phosphatidylethanolamine
PI: productively infected
PINK1: PTEN-induced putative kinase 1
RFP: red fluorescent protein
RNA: ribonucleic acid
ROS: reactive oxygen species
TBH: tert-Butyl hydroperoxide
TMRM: tetramethylrodhamine methyl ester
NLRP3: NLR Family Pyrin Domain Containing 3
NLRs: (NOD)-like receptors
IL-1β: Interleukin 1 beta
Casp-1: Caspase 1
CCCP: Carbonyl cyanide m-chlorophenyl hydrazone
SQSTM1/P62: Sequestosome-1
Δψm: mitochondrial membrane potential.
